# Towards quantitative point of care detection using SERS lateral flow immunoassays

**DOI:** 10.1007/s00216-022-03933-8

**Published:** 2022-02-03

**Authors:** Sian Sloan-Dennison, Emma O’Connor, James W. Dear, Duncan Graham, Karen Faulds

**Affiliations:** 1 Department of Pure and Applied Chemistry, Technology and Innovation Centre, University of Strathclyde, 99 George Street, Glasgow, G1 1RD UK; 2 The Queen’s Medical Research Institute, University/BHF Centre for Cardiovascular Science, University of Edinburgh, 47 Little France Crescent, Edinburgh, EH16 4TJ UK

**Keywords:** Lateral flow immunoassay, Point of care, Surface-enhanced Raman scattering, Portable spectroscopy

## Abstract

The rapid detection of biomolecules in a point of care (POC) setting is very important for diagnostic purposes. A platform which can provide this, whilst still being low cost and simple to use, is paper-based lateral flow immunoassays (LFIA). LFIA combine immunology and chromatography to detect a target by forming an immunocomplex with a label which traps them in a test zone. Qualitative analysis can be performed using the naked eye whilst quantitative analysis takes place by measuring the optical signal provided by the label at the test zone. There are numerous detection methods available; however, many suffer from low sensitivity and lack of multiplexing capabilities or are poor at providing POC quantitative analysis. An attractive method to overcome this is to use nanoparticles coated in Raman reporters as the labelled species and to analyse test zones using surface-enhanced Raman scattering (SERS). Due to the wide variety of metal nanoparticles, Raman reporter and laser excitations that are available, SERS-based LFIA have been adapted to identify and quantify multiple targets at once. Large Raman microscopes combined with long mapping times have limited the platform to the lab; however, by transferring the analysis to portable Raman instruments, rapid and quantitative measurements can be taken at the POC without any loss in sensitivity. Portable or handheld SERS-LFIA platforms can therefore be used anywhere, from modern clinics to remote and resource-poor settings. This review will present an overview of SERS-based LFIA platforms and the major recent advancements in multiplexing and portable and handheld detection with an outlook on the future of the platform.

**Graphical abstract:**

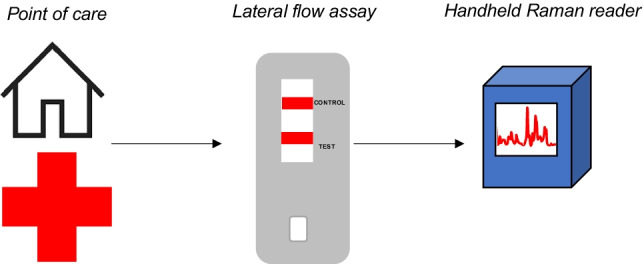

## Introduction

Lateral flow immunoassays (LFIA) are paper-based devices used for the rapid detection of analytes and biomolecules in clinical practice. The advantages of LFIA are exploited when the test is used in the community because the platform offers a simple-to-use and cheap-to-produce test that can be readily produced at scale. LFIA also have utility for use in hospitals; however, the specific application area and need must be thoroughly defined. For example, point of care (POC) assays based on multiple diverse platforms are being widely developed for a range of biomarkers that inform patient care pathways in hospitals and LFIA may not always be optimally suited. Where LFIA can have utility in hospitals are scenarios where rapidity and low cost are important test characteristics, such as screening for acute disease in emergency settings. The low cost of LFIA makes the platform well-suited for use in resource-poor settings such as diagnosis of infectious diseases in low-income countries. LFIA are also suited to these scenarios as they use smaller sample volumes and have fewer assay steps, shorter assay times and better storage conditions when compared to other detection techniques such as enzyme-linked immunosorbent assays (ELISA) and polymerase chain reactions (PCR). Before 2020, LFIA were probably best known at identifying pregnancy; however, they are now widely used to curb the spread of COVID-19 with 7.6 million tests taken in one month in England in 2021 alone [1]. 

The term ‘immunoassay’ refers to a recognition interaction, usually involving antibodies [2]. Commonly, a capture antibody and labelled detection antibody are used to capture target molecules in a sandwich immunoassay, binding with high affinity and specificity in a test zone on a nitrocellulose section of the LFIA strip. Typically, antibodies are labelled with spherical gold nanoparticles which are used as visual signal generators; however, other labels can be used including quantum dots, magnetic particles, fluorescence microspheres and latex beads. Using COVID-19 and pregnancy LFIA as an example, the test only needs to generate a qualitative result, i.e. a ‘yes/no answer’, and visual detection, based on the colour of the recognition label, is used as it produces a clear and easy-to-interpret result. The key point is that both COVID-19 and pregnancy LFIA detect protein targets that are not present in biofluids of unaffected individuals. However, when detecting molecules whose concentration can be indicative of a disease, a sign of infection, an unsafe level of hazardous material in an environmental sample or food contamination, it is vital that the LFIA yield quantitative information which can be achieved by externally measuring the amount of label present at the test zone and relating this to the concentration of the molecule [3]. Depending on the label, quantitative analysis can be achieved using colorimetric, fluorescence, electrochemical, and surface-enhanced Raman scattering (SERS) detection.

## Raman mapping SERS-LFIA platform

**Fig. 1:**
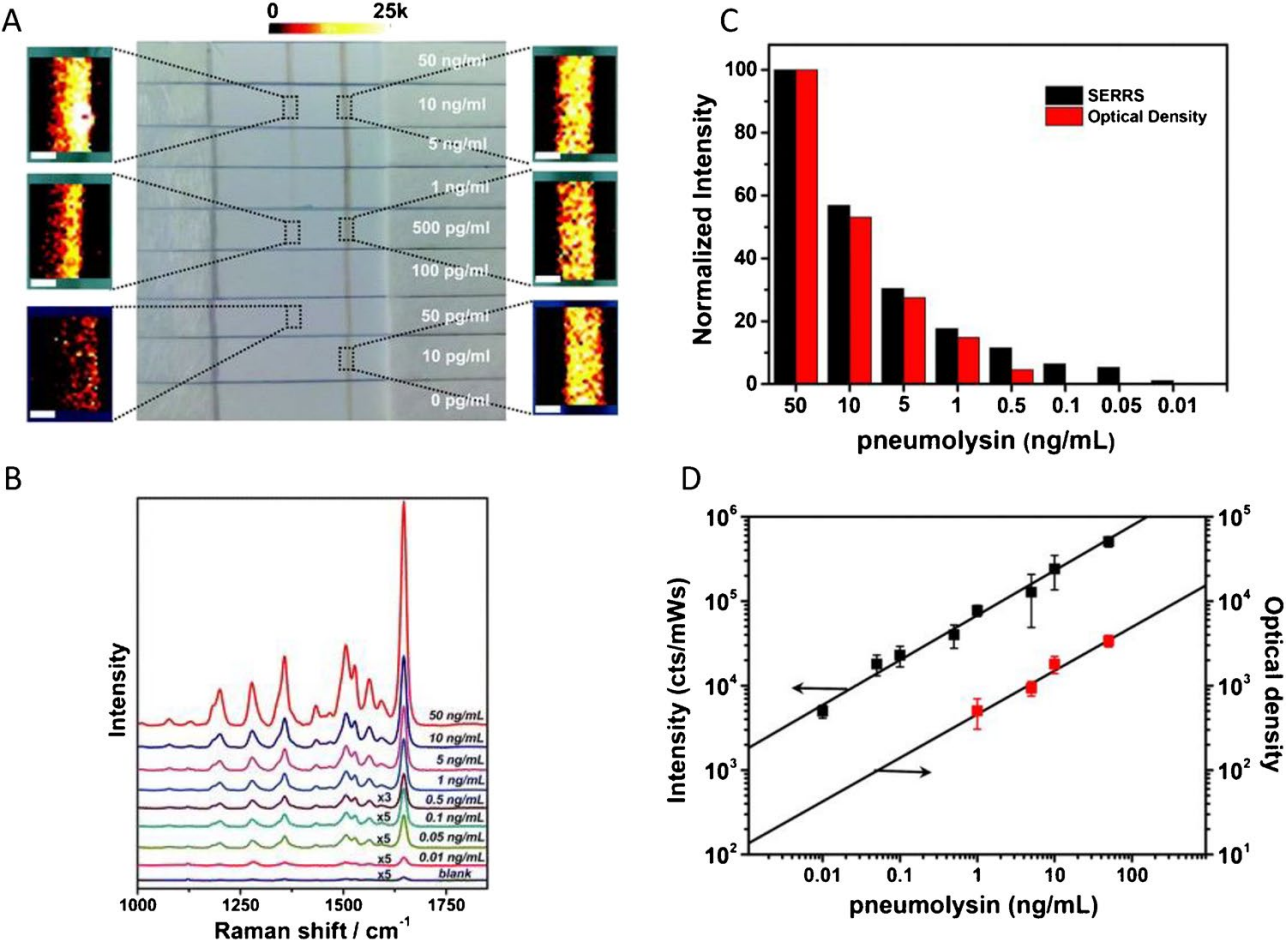
**A** Photographs of different LFIA strips run with different pneumolysin concentrations as indicated. SERRS maps using the 1647 cm^−1^ band of MGITC. **B** Average SERRS spectra from 50 random points measured in the test lines. **C** Evaluation of the results obtained for different pneumolysin concentrations using the point of care-based LFIA (red columns) and SERS-based LFIA (black columns) through optical density and SERS measurements, respectively. **D** Quantification range for pneumolysin employing the optical LFIA strip (red squares) or the SERS-based LFIA strip (black squares). The bars represent the standard deviation from three independent measurements [6]. Reproduced from Ref. 6 with permission from the Royal Society of Chemistry

SERS is a vibrational spectroscopy technique used to enhance weak Raman scattering signals by adsorbing a molecule onto a roughened metal surface. Typically, gold and silver nanoparticles are used and enhancement factors of up to 10^10^ have been achieved [4]. To incorporate SERS analysis into a LFIA, the detection nanoparticle is labelled with a Raman reporter and a recognition moiety that is specific to the molecule of interest, most commonly an antibody. The Raman reporter should be a molecule with a large Raman scattering cross section and contain sulphur or nitrogen atoms due to their high affinity for gold and silver which allows the reporter to be close to the nanoparticle surface and experience SERS enhancement due to the electromagnetic effect [5]. For further enhancement, Raman reporters with absorption spectra matching the excitation wavelength can induce surface-enhanced resonance Raman scattering (SERRS) which can lead to increased sensitivity. When the LFIA assay is run to build a calibration or limit of detection (LOD) curve, the test zone, which could be a spot or line, can be analysed using a Raman spectrometer at an appropriate laser excitation. Conventionally, the analysis of SERS-LFIA has taken place by Raman mapping the test line of the LFIA strips using large Raman microscope systems. The decrease in SERS signal with decreasing concentration of target molecule present can be visualised by creating heat maps of the signal across the test line. Quantitative analysis is achieved by averaging the SERS signal from the entire map and plotting the intensity of one of the main peaks of the Raman reporter against the concentration of the target molecule. An example of this approach used for the detection of pneumolysin using gold nanoparticles coated in a detection antibody and the Raman reporter malachite green isothiocyanate (MGITC) is shown in Fig. 1 [6]. The results also indicated that the SERS analysis was more sensitive than optical measurements.

The Raman mapping approach to SERS-LFIA has been used to detect a number of different clinically relevant biomolecules using different nanoparticle and Raman reporter combinations. For example, gold nanoparticles labelled with MGITC have been used to detect a myriad of biomolecules including HIV-1 DNA [7] and thyroid-stimulating hormone [8]. In these examples, a single biomolecule was detected on each test zone. However, the simultaneous detection of different biomolecules from one sample could be highly beneficial and has several benefits including improving the efficiency of testing, reducing costs and potentially leading to better patient stratification.

## Multiplex biomarker detection platforms

### Spatially resolved detection

**Fig. 2:**
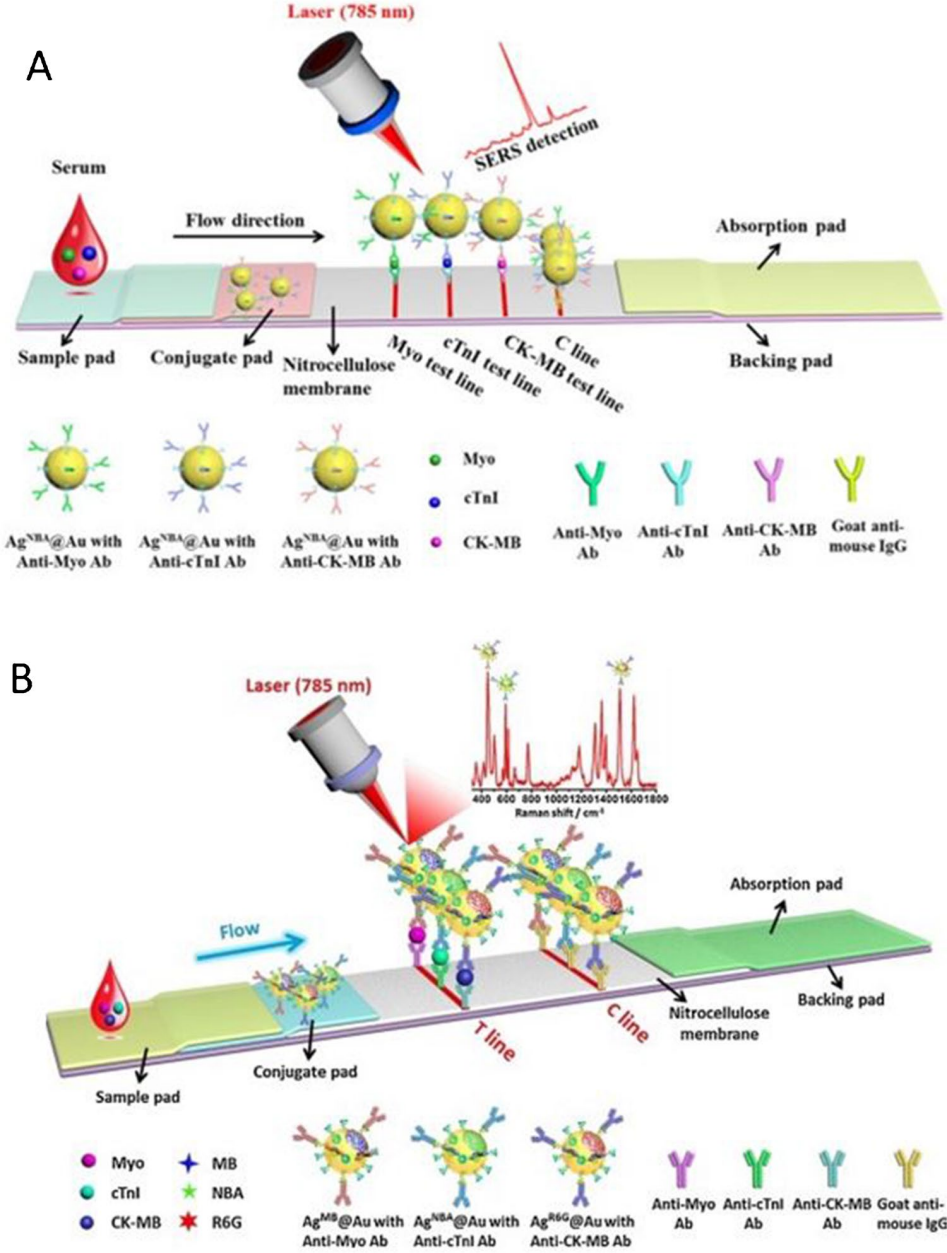
**A** Schematic illustration for the detection of three cardiac biomarkers using SERS-LFIA in a spatially resolved format [13]. **B** Schematic illustration for the detection of the same three cardiac biomarkers using SERS-LFIA in a signal-resolved format [17]. Panel **A** reprinted from *Biosensors and Bioelectronics*, 106, Zhang, D,; Huang, L.; Liu, B.; Ni, H.; Sun, L.; Su, E.; Chen, H.; Gu, Z.; Zhao, X., Quantitative and ultrasensitive detection of multiplex cardiac biomarkers in lateral flow assay with core–shell SERS nanotags, 204–211, 2018, with permission from Elsevier. Panel **B** reprinted from *Sensors and Actuators B: Chemical*, 277, Zhang, D.; Huang, L.; Liu, B.; Su, E.; Chen, H.-Y.; Gu, Z.; Zhao, X., Quantitative detection of multiples cardiac biomarkers with encoded SERS nanotags on a single T line in lateral flow assay, 502–509, 2018, with permission from Elsevier

Spatially resolved multiplexed detection involves the placement of multiple test lines, or zones, on a single LFIA device. This allows the detection of multiple biomolecules simultaneously on one test strip. In some instances, the label for each biomolecule can be the same, in which case the spatial location differentiates the biomarker; however, this method has also been investigated using multiple labels that give a specific response depending on the analyte they are designed to detect. Raman mapping SERS-LFIA platforms have been employed for spatially resolved duplex multiplexed detection. For example, Kaposi’s sarcoma-associated herpes virus and bacillary angiomatosis [9], antibiotics neomycin and quinolones [10], *Listeria monocytogenes* and *Salmonella enterica* serotype *Enteritidis* [11], and influenza A H1N1 virus and human adenovirus have all been detected simultaneously from separate test zones on the same strip [12]. Similarly, triplex detection has been achieved using SERS-LFIA. Zhang et al. detected three cardiac biomarkers (myoglobin, cardiac troponin I and creatine kinase-MB isoenzymes) on a single LFIA [13]. A schematic of the LFIA showing the three test lines consisting of the capture antibodies for each biomolecule and gold nanoparticles coated in the Raman reporter and the relevant detection antibodies is shown in Fig. 2A. This type of analysis was taken even further by Zhang et al. who developed a SERS-based microarray on a LFIA that was capable of the detection of 11 nucleic acids associated with different respiratory tract infection pathogens [14]. On five of the test zones, two different capture nucleic acids were spotted whilst one zone had only one capture nucleic acid. Using two different Raman reporters and 11 different detection nucleic acids, the assay was built so that the test zones containing capture nucleic acids specific to different targets immobilised two detection nanoparticles each made with the different Raman reporters. Therefore, the SERS signal from this spot produced a duplex SERS spectrum from the mixture of both reporter signals. If both targets were present, features from both reporter spectra would be observed [14]. This final example of spatially resolved multiplexed detection also incorporates signal-resolved detection by the use of a duplex SERS signal from a single test zone. Whilst the capabilities of spatially resolved multiplexing have been well demonstrated, this approach has some disadvantages. For example, the number of test lines that can be incorporated into a single device is finite and having an increasing number of test regions will undoubtedly increase the overall analysis time. There is an option to incorporate an architecture change of the device to make it longer; however, this would increase the flow time of the device, thereby increasing the time of the test. Because of these concerns, signal-resolved detection has been attempted where the detection of multiple analytes from a single test zone is attainable.

### Multiplexing from signal-resolved detection

Signal-resolved detection occurs when multiple labels are immobilised at a single test zone and can be identified through their specific signals generated from the label. The key factor is that all signals are collected from the same region simultaneously, offering rapid analysis and return of results. With regard to LFIA devices, this also means no deviation from the traditional device architecture which would incur increased materials costs. Signal-resolved multiplexing on LFIA has been achieved with colorimetric analysis using orange, red and green silver nanoparticles for the detection of different viruses. However, the colour of the mixed signal labels can be difficult to interpret at low concentrations. Fluorescence spectroscopy also struggles in a multiplexing capacity due to its broad emission bands which have large spectral overlap making the labels difficult to deconvolute. SERS is ideal for signal-resolved multiplex detection as it benefits from the fact that a range of different Raman reporters exist, generating characteristic, ‘fingerprint’ spectra specific to the Raman reporter. If a number of different Raman reporter-labelled nanoparticles are mixed together in a sample, distinct peaks from each of their individual spectra can be picked out of the multiplexed spectrum generated from the mixture and the individual reporters can be identified [15]. In this way, different reporters can be used to represent multiple analytes in a single sample. The presence of characteristic peaks of Raman reporter in the multiplex spectrum can be used for qualitative analysis and the intensity of the peak can be used to gain quantitative information.

In the context of LFIA, SERS multiplexing has been used to detect multiple biomolecules at once. For example, Wu et al. used gold nanoparticles functionalised with two Raman reporters for the duplex detection of *L. monocytogenes* and *S. typhimurium* respectively on a single test zone. Quantitative information was obtained based on decreasing intensity of the peaks from the SERS signal of the Raman reporter present in the multiplex test zone spectrum, with decreasing concentrations of the targets [16]. Zhang et al. used three Raman reporters to code for the presence of cardiac biomarkers myoglobin, cardiac troponin I and creatine kinase-MB isoenzymes respectively in a signal-resolved detection assay [17]. A schematic of this is shown in Fig. 2B depicting the mixed capture antibody test line and three gold nanoparticles coated with the relevant detection antibody and three different Raman reporters. The authors achieved low detection limits, well within the clinical ranges of each biomarker. Sanchez-Purra et al. achieved duplex detection of dengue and zika virus using SERS [18]. Their dipstick LFIA platform was also capable of quantitative analysis. In a different publication, the same researchers demonstrated proof-of-concept study showing that a pentaplex SERS signal could be obtained from a single test region [19]. A generic capture protein was immobilised on the test region of the LFIA device and the control line consisted of a second capture protein. Peaks from each of the individual SERS spectra were clearly visible in the pentaplex spectrum, therefore implying that signal-resolved pentaplex detection can be achieved using SERS, although quantification was not demonstrated.

The advantages of signal-resolved multiplexed detection include rapid analysis for the detection of multiple analytes from a single sample; therefore, the results offer more information in the time it takes to run a single LFIA device. Smaller quantities of reagents are used both in the preparation and in the running of the device and the device architecture does not need to be drastically changed, lowering the cost associated with materials. However, the immunoreactions taking place must be highly specific and ensure cross-reactivity is at a minimum. Depending on the analysis method, this approach may also be limited to the number of signals that can be generated from a single region and how these can be separated. This is why SERS offers the greatest potential for signal-resolved multiplexing as a range of Raman reporters can be used.

In these examples, the SERS-LFIA analysis was shown to offer superior sensitivity and multiplexing capabilities and produce quantitative information when compared to other detection methods. However, the pursuit of achieving the most sensitive SERS-LFIA is all in vain if the analysis is limited to benchtop microscope systems in the laboratory to get the necessary readout. LFIA are designed to be rapid, used at the POC and a new portable and handheld SERS ‘readout’ method is vital to combine both the merits of sensitive SERS and portable and rapid LFIAs. By performing the SERS analysis on portable Raman spectrometers, the SERS-LFIA can be transferred out of the lab to a number of different POC environments, providing rapid, sensitive and quantitative information for many different, clinically relevant biomolecules in real time.

## Portable SERS-LFIA platform

The simplest way to transfer SERS-LFIA analysis to POC, with no loss in sensitivity, is to use a portable Raman spectrometer. In this setup, the laser can be focused onto the test zone using microscope with a 5 or 20 × objective or using a fibre optic probe, and then, a number of scans can be taken to produce an average SERS response. This platform has been applied for the detection of rotavirus [20], S-100β protein [21], microRNA-21 [22], serum amyloid A and C-reactive protein [23], and IgM/IgG covid antibodies [24]. This setup has also been used in a multiplexing capacity for the detection of 6 major mycotoxins in maize, utilising dual Raman reporters and triple test lines [25]. However, the inclusion of a Raman microscope or fibre optic stand has a few disadvantages. These include reducing the portability as it may need to be set up in a central area near a POC setting, for example in a lab at the hospital and the strips taken to the instrument, therefore increasing the time until results. Whereas if a handheld instrument was used, the analysis could take place next to a patient. The microscope also increases the cost of the platform as microscope systems can be expensive. Also, the platform is less user friendly as training would be needed to focusing the laser beam, whilst the open beam could also present a safety issue.

**Fig. 3:**
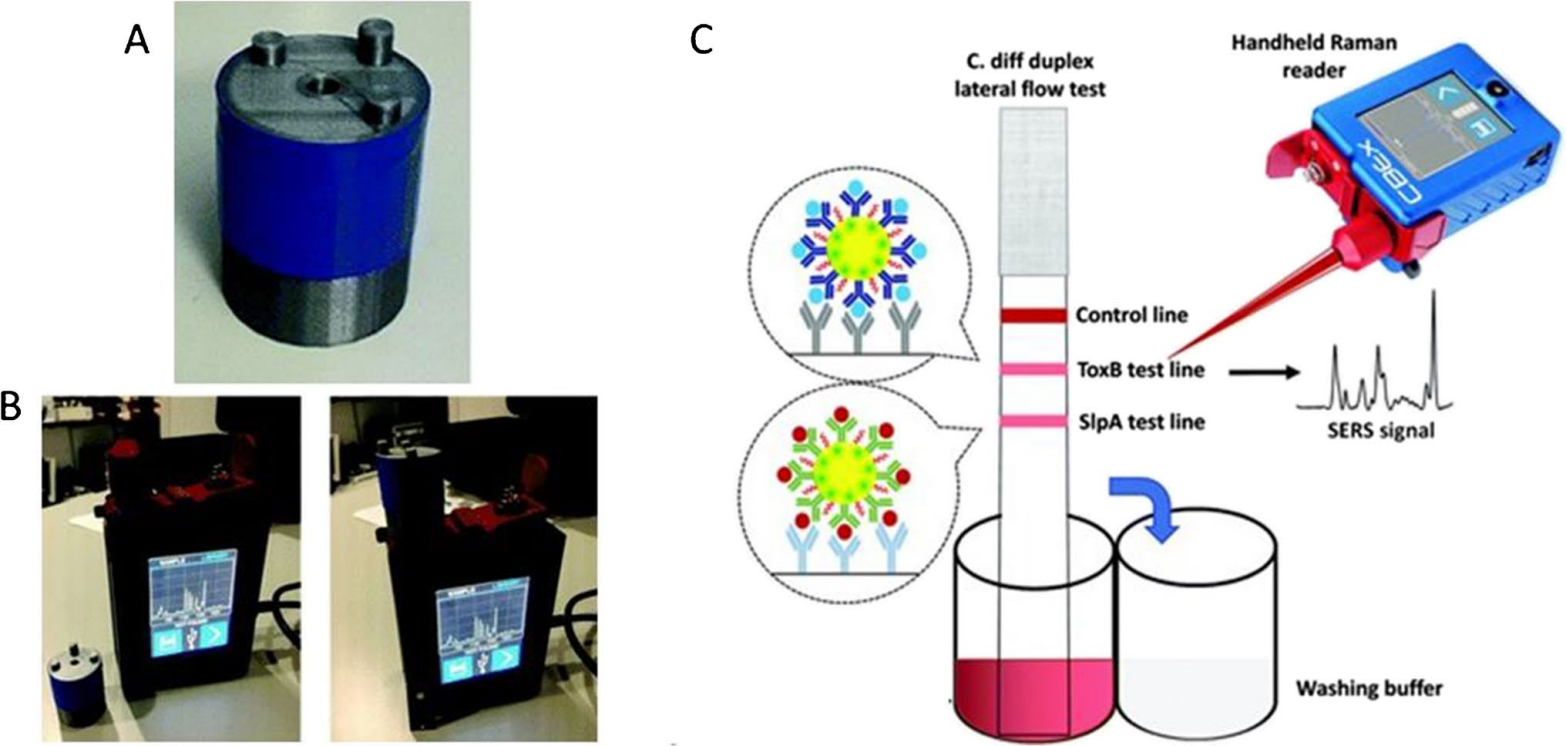
**A** Image of 3D printed adaptor [26]. **B** Image displacing how adaptor fits over point and shoot lens [26]. **C** Schematic representation of the spatially resolved detection of *clostridium difficile* using point and shoot handheld Raman spectrometer [27]. Reproduced from Refs. 26 and 27 with permission from the Royal Society of Chemistry

The microscope can be removed from the platform and the point and shoot feature of many portable Raman spectrometers can be exploited by holding the laser aperture directly above the test line of the LFIA strip and then taking a reading, thus removing the need for the microscope. This creates a ‘handheld’ approach which can be used directly next to the LFIA user. Taking into account the focal distance of the laser, an inexpensive 3D printed adapter, which holds the strip in front of the laser at the correct focal distance, can be used to give reproducible results. The adapter was first developed for the integration of SERS analysis and paper-based microfluidic devices for the detection of miR-29a [26]. A photograph of the adapter and how it fits over the point and shot lens of a handheld Raman spectrometer is shown in Fig. 3A and B. This platform has since been used for SERS analysis of LFIA by Hassanain et al. who demonstrated the selective, sensitive, rapid and cost-effective diagnosis of *clostridium difficile* infection which was achieved using spatially resolved multiplex detection of two specific biomarkers [27]. A schematic of the LFIA and point and shoot platform is shown in Fig. 3C. Smartphone-based Raman spectrometers equipped with a 5G cloud-based healthcare management system have also been applied for point and shoot analysis for the quick, on-site diagnosis of sepsis [28]. In these examples, point scanning was carried out using a laser spot which could compromise the working efficiency as it only analyses a limited fraction of the test zone, which can lead to under sampling. Orbital raster scans and multiple acquisitions along the test line can increase the reproducibility, as could line illumination [29].

**Fig. 4:**
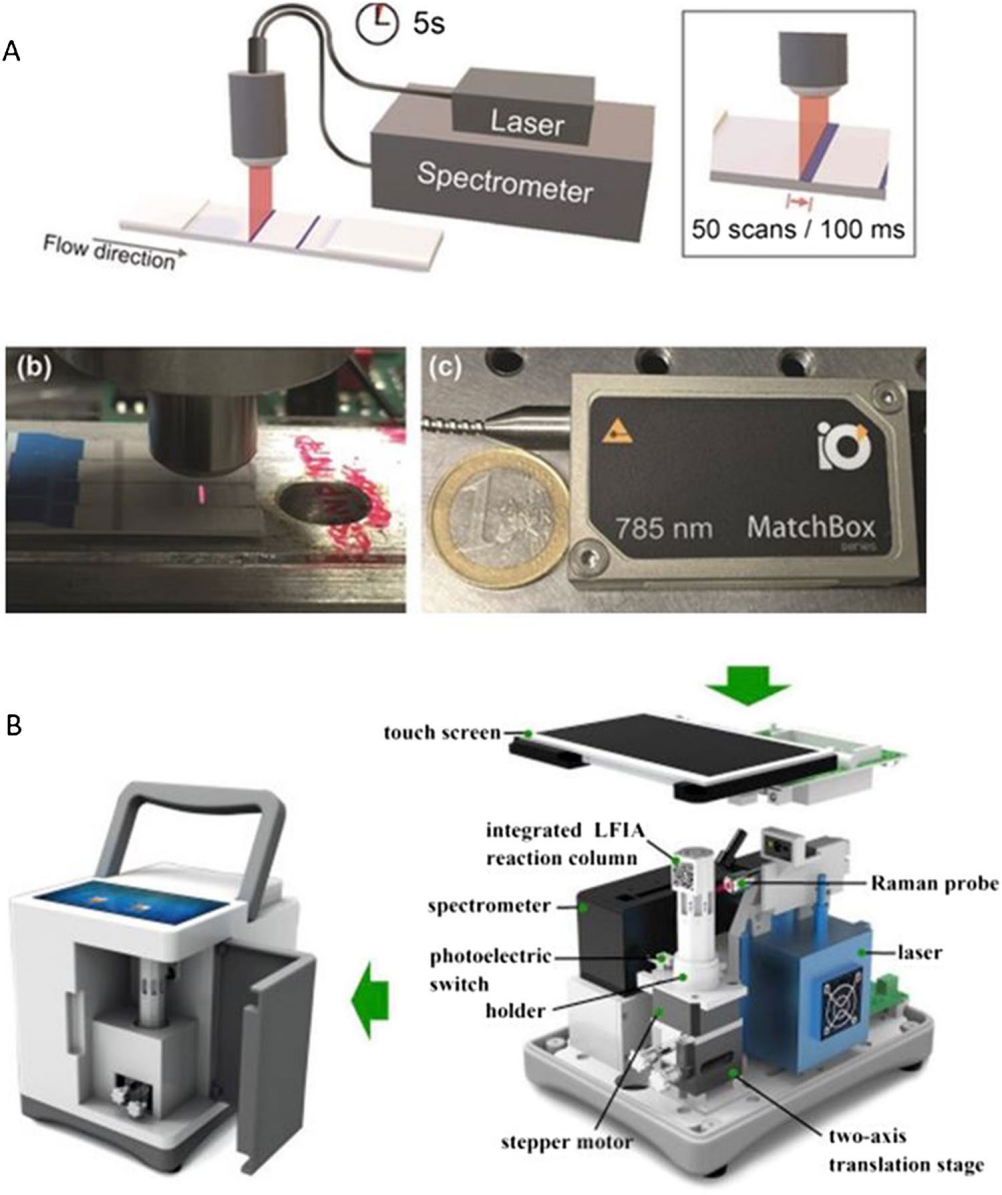
**A** Schematic of the portable SERS-LFIA detector system with line illumination [30]. **B** Inside and outside all-in-one portable SERS-LFIA platform [33]. Panel **B** reprinted from *Biosensors and Bioelectronics*, Xiao, R.; Lu, L.; Rong, Z.; Wang, C.; Peng, Y.; Wang, F.; Wang, J.; Sun, M.; Dong, J.; Wang, D.; Wang, L.; Sun, N.; Wang, S., Portable and multiplexed lateral flow immunoassay reader based on SERS for highly sensitive point-of-care testing, 112,524, 2020, with permission from Elsevier

A SERS-LFIA reader which addresses issues of under sampling was developed by Tran et al. for the rapid, quantitative and ultra-sensitive detection of the pregnancy hormone human chorionic gonadotropin [30]. They identified the major drawback of current SERS-LFIA as being the long readout time due to the Raman mapping analysis and developed a portable SERS reader combined with a motorised stage for rapid scanning of the test strip. The setup is shown in Fig. 4A. The average SERS signal of the test line was achieved using line illumination to scan orthogonally to the test line using the stage, which moved the strip through the laser focus. Fifty positions, including both on and off the line, were scanned with an overall analysis time of 5 s, several orders of magnitude faster than mapping. The line illumination, compared to the conventional spot illumination, also assured that as much of the test line was measured as possible, with no information from the line being lost. The setup was also used to detect the SERS signal from 5 different Raman reporter-coated nanoparticles from a single test line in a proof-of-concept detection of protein A. This demonstrated the platform had multiplexing potential for the simultaneous detection of multiple biomolecules. The compact reader has now been applied for the detection of SARS-CoV2-specific IgM and IgG antibodies [31]. The setup was slightly modified to rapidly scan both the test and control line using a mechanical sample holder. The compact reader was able to detect both antibodies when conventional naked eye detection showed no visible test line. Jia et al. have also incorporated a stage in their portable SERS-LFIA platform; however, this system was automated and uses software to control the movement of the lead screw motor to automatically and randomly detect multiple point of the test zone [32]. This reduced the detection error caused by operation by non-professionals and could sensitively detect west Nile virus, 100-fold more sensitive than visual analysis. Although necessary for the scanning or automatized analysis, the inclusion of the external stage could also limit the portability aspect. Using a mechanical, instead of a motorised, stage does limit this problem and make the system more accessible. However, there is also the issue of an open laser beam which is a major safety risk for POC environments. To increase the likelihood of SERS-LFIA platforms progressing to approval for POC, a compact, handheld all-in-one system is desirable.

## All-in-one SERS-LFIA platform

An all-in-one enclosed ‘handheld’ platform which allows the analysis of LFIA strips to take place directly next to the laser source, whilst maintaining safety measures, is vital if SERS-LFIA is ever to be recognised as a POC analysis method. The first steps to achieving this have been reported by Xiao et al. who used a SERS portable reader integrated with a LFIA reaction column (33). Their design and enclosure are shown in Fig. 4B. They hypothesised that the major disadvantage of conventional SERS analysis is the expensive, large spectrometers which can only record the SERS spectrum at one position of the LFIA test strip at a time, and that multiple measurements require the strip to move on the xy translation stage repeatedly. Taking this into account, their proposed reader integrated a LFIA column which was mounted onto a step motor which can be moved up, down, back and forth by adjusting a two-axis translational stage allowing detection of both the test and control lines using a Raman probe with a laser spot of 200 µm. The automated SERS-based lateral flow system was housed in an enclosure with a handle to move the instrument, allowing the SERS-LFIA platform to be brought to the POC.

## Outlook

Combining SERS with LFIA adds the ability to quantify biomarker concentration in POC settings. However, the reader needs to be easy to use and provide the patient and/or clinician with easy-to-interpret actionable results. In healthcare systems, SERS-LFIA would allow measurement of biomarkers rapidly and cheaply out with current settings, for example in family doctor practices, ambulances and pharmacies. There is also a drive to decentralise clinical drug trials and facilitate data collection in patient’s homes. This promises to make trials more inclusive, cheaper and potentially safer (early detection of drug toxicity, for example). SERS-LFIA is suited to this new application, where it could be used to monitor biomarkers related to drug toxicity, providing the assay and reader can be optimised for home use. In low-income settings, SERS-LFIA could be very valuable to allow immediate decision-making as the availability of tests is often limited, especially in rural settings. The characteristics of the assay for use in low-income settings such as parts of Africa will be different to those required for home use in Western countries. Key aspects of the target product profile will include resistance to weather conditions (temperature, humidity) and minimal storage requirements, components which can be manufactured locally, low production cost, and results that provide clinicians with actionable information within a specific, locally relevant, patient care pathway.

However, to date, the majority of research reported so far has been confined to laboratory-based SERS-LFIA due to using benchtop instruments to carry out Raman mapping analysis to obtain average signals across the lateral flow strips. Therefore, the development of portable Raman spectrometers, combined with a suitable sampling accessory and optics to average and maximise the signal readout across the lateral flow strip, is required to move SERS-LFIA detection platforms to the POC. The sampling accessory can limit the portability and stability of the system and if SERS-LFIA is to become a routine LFIA analysis technique used in a clinical setting, all-in-one, handheld, Raman systems need to be developed which allow the LFIA strip and SERS measurements to take place in an enclosed system. This will provide rapid results that are produced next to the patient leading to much faster decision-making in urgent care situations. Enclosed sampling accessories will also mitigate laser safety issues that can occur with open beam microscope and stage systems. All-in-one systems have previously been published, and are in current development, but these tend to use spot illumination. However, in order to obtain high sensitivity measurements and maximise signal readout across the whole LF strip, this method needs to be combined with line illumination to avoid under sampling of the strip, allowing for an ultra-sensitive handheld SERS-LFIA platform.

Another issue that needs to be addressed when using SERS outside the confines of a controlled laboratory environment is the ability to accurately detect the concentration of a biomolecule in a complex biological sample without having to build a calibration model. In an ideal POC situation, the sample will be run on the LFIA, the strip analysed within 20 min, and the intensity of the SERS signal as read will be directly related to the concentration. This requires a prior calibration model to be built that takes into account the sample component matrix and mitigate against any background interference from the sample matrix. Therefore, data analysis algorithms are required which will relate the SERS signal to the concentration, but also produce an easily interpretable result for the end user who will not be a spectroscopist. Although achievable, the use of nanoparticles and biological matrices can result in aggregation on test lines which can give artificially high SERS signals due to hotspot formation and could give false, high concentration results. Careful consideration into nanoparticle and Raman reporter stability and shelf life is key to obtaining reproducible results, along with control measures such as calibrating against the control line which could also be implemented to standardise the results. Although there are obstacles that need to be overcome, the sensitive, multiplexing, portable technology that this platform can achieve is worth pursuing in order to develop a gold standard handheld SERS-LFIA for the rapid, absolute quantification of clinically relevant biomolecules at the POC. Overcoming these challenges is achievable and we believe that SERS-LFIA will be used at the POC within the next few years.
